# A novel missense variant of HS6ST2 gene in Paganini-Miozzo syndrome with a rare neurodevelopmental and Endocrine phenotypes

**DOI:** 10.3389/fgene.2025.1515260

**Published:** 2025-07-04

**Authors:** Meiling Zhang, Xiao Chang, Xiaoyun Du, Zhen Chen, Xinyue Zhang, Fucheng Cai

**Affiliations:** ^1^ Department of Pediatrics, Union Hospital, Tongji Medical College, Huazhong University of Science and Technology, Wuhan, China; ^2^ BGI Genomics, Shenzhen, China; ^3^ Clinical Laboratory, BGI Genomics, Wuhan, China

**Keywords:** Paganini-Miozzo syndrome (MRXSPM), HS6ST2, whole exome sequencing (WES), developmental delay, endocrine abnormality

## Abstract

Paganini-Miozzo syndrome (MRXSPM) is a globally rare disease caused by hemizygous mutations in the *HS6ST2* gene on chromosome Xq26. This study presents the first case of MRXSPM in China, meanwhile the fourth case worldwide. The proband was admitted to the hospital due to developmental delay. Whole exome sequencing (WES) revealed a novel variant, c.764C>A (p.Pro255Glu) missense mutation in the *HS6ST2* gene. Brain CT showed mild lateral ventricular enlargement, and electroencephalogram showed diffuse spikes and waves. Biochemical tests indicated significantly elevated transaminases, blood lactate values, and lactate/pyruvate values. Bioinformatics predictions suggest that this mutation may affect the thermal stability of the HS6ST2 protein. The amino acid where the mutation c.764C>A p.P255Q occurs is conserved across multiple species, specifically being proline in 13 species. *In vitro* cell experiments demonstrated that this mutant can impact the expression of HS6ST2 protein at post-transcriptional level. Comparison with previously reported cases revealed that different mutations might lead to different alternations in the function of HS6ST2 protein, resulting in distinct clinical phenotypes.

## 1 Introduction

Paganini-Miozzo syndrome (MRXSPM) is a neurodevelopmental disorder characterized by global developmental delay, impaired intellectual development, high myopia, and mild facial deformity (Paganini et al., 2019). The syndrome is caused by a hemizygous mutation in the *HS6ST2* gene, which is located on Xq26 and encodes heparan sulfate 6-O-sulfotransferase 2 (HS6ST2). HS6ST2 is a member of the HS6ST family, which comprises three subtypes (Habuchi et al., 2000). The primary role of HS6ST is to catalyze the addition of sulfate to heparan sulfate proteoglycan (HSPG), thereby playing a crucial role in cell growth, differentiation, adhesion, and migration (Song et al., 2011; Forsten-Williams et al., 2005). Previous studies have reported that HS6ST2, as a member of the HS6ST family, is associated with tumor occurrence and visual development (Jin et al., 2017; Cai et al., 2014). To date, only three confirmed cases of MRXSPM have been reported globally. Among these cases, missense mutations were identified in the *HS6ST2* gene located on chromosome Xq26.2 (Paganini et al., 2019; Sarmadian et al., 2023). Specifically, two identical twins from Italy exhibited a hemizygous single nucleotide variant c.916G>C (p.Gly306Arg) in the *HS6ST2* gene, while the third case involved an Iranian child with the *HS6ST2* gene variant c.979C>T (p.Pro327Ser) (Paganini et al., 2019; Sarmadian et al., 2023). In this study, we present the fourth documented patient with MRXSPM worldwide, and the first patient diagnosed with this condition in China. Utilizing whole-exome sequencing, we identified a novel mutation site, c.764C>A (p.Pro255Glu), on the *HS6ST2* gene.

In this study, we conducted a comprehensive analysis of the clinical phenotypes of the study patients. Furthermore, we performed bioinformatics analysis on the c.764C>A (p.Pro255Glu) site. The results revealed that this mutation site is highly conserved and has a significant impact on the thermal stability of the HS6ST2 protein, leading to a reduction in its catalytic activity. Additionally, our *in vitro* cell experiments demonstrated that this mutation can impact the expression of HS6ST2 protein at the post-transcriptional level. This effect may be attributed to the mutation causing alterations in the rate of protein degradation.

## 2 Materials and methods

### 2.1 Samples, ethic approve and consent to participate

The samples used in this study were peripheral blood obtained from proband and their parents. The study received ethical approval from the Ethics Committee of Union Hospital Affiliated to Tongji Medical College of Huazhong University of Science and Technology (license no. 20230880). Informed consent forms for WES analysis were signed by the parents of the proband, specifically for the purpose of this research.

### 2.2 Whole exome sequencing (WES)

Genomic DNA was extracted from peripheral blood samples of the patient and his parents according to the manufacturer’s instructions (MagPure Buffy Coat DNA Midi KF Kit, MAGEN). Genomic DNA was fragmented using the Covaris S220 Focused Ultrasonicator (Covaris, Woburn, MA, United States), AMPure XP Beads (Life Sciences, Indianapolis, IN, United States) were used to select 200–300 bp fragments to construct a DNA library. Exome capture using the MGIEasy Exome Capture V4 probe (MGI) was followed by paired-end read sequencing (2 × 100 bp read length) on the MGISEQ-2000 platform. Raw reads were aligned to the human genome reference (hg19) using the BWA (Burrows Wheeler Aligner). GATK UnifiedGenotyper (https://www.broadinstitute.org) was used to detect single-nucleotide variants and small insertion or deletion variants. Possible copy number variations were analyzed using ExomeDepth. Variants were classified according to the American College of Medical Genetics and Genomics (ACMG) Variation Interpretation guidelines.

The mammalian HS6ST2 cDNA (NM_001077188.1, ENST00000521489.5) was used as template to generate a HS6ST2 c.764C>A mutant sequence, by site-directed mutagenesis fused to pCMV-3×FLAG-Neo eukaryotic expression vector. Primers used to obtain the C>A nucleotide substitution were as follows: Forward, 5′

- ATC​CAG​CTG​GAG​CAG​CAG​TGC​GAG​TGC​CGC​GTG-3′ and Reverse, 5′
- CAC​GCG​GCA​CTC​GCA​CTG​CTG​CTC​CAG​CTG​GAT-3’. Primers used to obtain wild type HS6ST2 were as follows: Forward, 5′
- GCT​CGA​ATT​CGC​CAC​CAT​GGA​CTA​CAA​AGA​CCA​TGA​CGG-3′ and Reverse, 5′
- TCG​AGA​TAT​CTT​AAC​GCC​ATT​TCT​CTA​CAC-3′.

### 2.3 Bioinformatic analysis

PolyPhen-2 (http://genetics.bwh.harvard.edu/pph2/index.shtml) was applied for the conservation analysis of genetic mutations. At the protein level, the differences between the p.Pro255Glu substitution and wild type in the secondary structure were analyzed by SOPMA database (https://npsa-prabi.ibcp.fr/cgi-bin/npsa_automat.pl? page = npsa_sopma.html). The consequences of the p.Pro255Glu substitution on HS6ST2 thermal stability were evaluated using DUET (http://biosig.unimelb.edu.au/duet/stability).

### 2.4 Cell culture and transfection

HEK-293T cells were grown in Dulbecco’s modified Eagle’s medium (DMEM) supplemented with 10% fetal bovine serum (FBS) and 1% 100 μg/mL penicillin-streptomycin (BL505A, Biosharp) at a temperature of 37°C in a 5% CO_2_ atmosphere.

HEK-293T cells were seeded onto 6-well plate at a density of 4.0 × 10^5^ and grown to the confluence reaching about 80% at the time of transfection. Cells were transfected with 2 µg of pCMV-HS6ST2-Mut or pCMV-HS6ST2-WT using lipofectamine™ 3000 (L3000008, Invitrogen, CA) for 24 h, and were serum-starved and grown for additional 24 h before being harvested. In the transfection experiments, appropriate vector DNA was used as a negative control and used to ensure similar DNA concentrations in all conditions.

### 2.5 Quantitative RT-PCR (qRT-PCR)

Total RNA was extracted with TRIzol reagent (9109, TaKaRa) according to the manufacturer’s instructions. RNA was reverse transcribed using reverse transcription kit (11123ES70, YEASEN). The gene expression levels of mRNA’s were measured by SYBR Green Realtime PCR Master Mix (QPK-201, Toyobo). GAPDH was used as the internal control. The calculation of mRNA’s relative repression levels was carried out using the 2^−ΔΔCt^ method. The following primers were used in this study: HS6ST2 Forward, 5′-CTT​CGG​CCT​CAC​TGA​GTT​TC-3′ and Reverse, 5′-CCT​CCT​GAT​GCT​CTT​TCT​GC-3′; ACTIN Forward, 5′-TGA​CGT​GGA​CAT​CCG​CAA​AG-3′ and Reverse, 5′-CTG​GAA​GGT​GGA​CAG​CGA​GG-3′.

### 2.6 Western blot

Total protein was extracted from HEK-293T cells for Western blot. Following primary antibodies were applied in this study: mouse anti-flag antibody (GS20002A1170, Mabnus), rabbit anti-GAPDH antibody (2118S, CST). The protein signals were detected using Pierce™ ECL Western blotting substrate (SQ202, YAMEI). Gray density of protein band was measured by using ImageJ software.

### 2.7 Statistical analysis

The results were presented as the mean ± s.d. The t-test was used to compare the means of two groups. *P* < 0.05 was considered statistically significant. All statistical analysis was performed with GraphPad Prism 9.0.

## 3 Results

### 3.1 Proband

**FIGURE 1:**
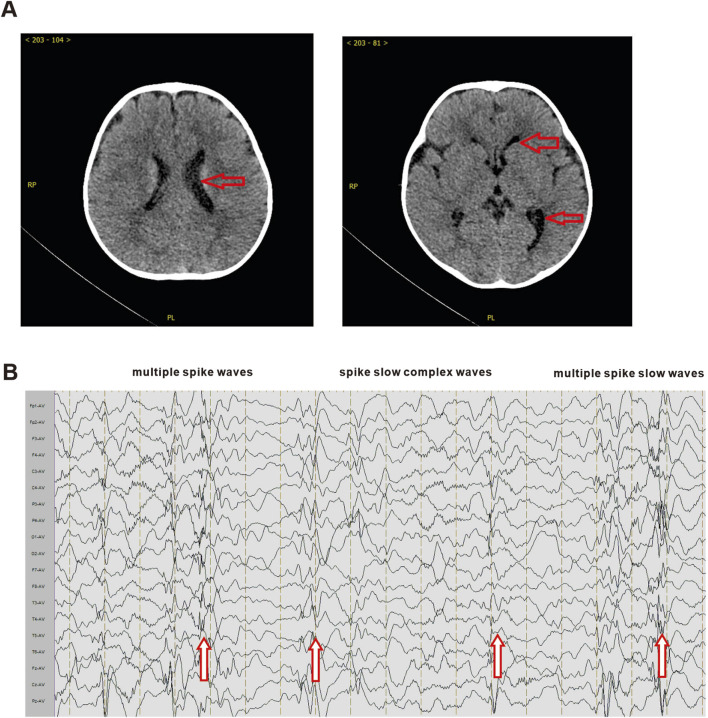
Brain CT and electroencephalogram of proband. **(A)** The brain CT scan results of the prband. Red arrows indicate the enlarged left lateral ventricle. **(B)** The EEG results of the proband. Red arrows indicate multiple spike waves, spike slow complex waves and multiple spike slow waves.

A 9-month-old male was admitted to the Union Hospital Affiliated to Tongji Medical College of Huazhong University of Science and Technology in Nov. 2021, with complaints of growth retardation for 3 months. He had been delivered at full term after 40 weeks of gestation (birth weight 4,100 g, Apgar score not clear), without birth trauma, asphyxia, or pathological jaundice. and was the first child of his mother. The patient’s parents are healthy, non-consanguineous, and have no family history or genetic metabolic history. The results of biochemical test revealed a poor liver function, with alanine aminotransferase 421 U/L (normal range 5–40 U/L); aspartate aminotransferase 71 U/L (normal range 8–40 U/L). Additionally, the blood tests indicated high levels of blood ammonia (135 μmol/L, normal range 9–30 μmol/L) and blood lactate (22.9 mg/dL, normal range 4.5–20 mg/dL). However, no significant abnormalities were observed in other biochemical tests. Ultrasound examinations of the liver, gallbladder, pancreas, and spleen showed no obvious abnormalities. Brain CT scan revealed a wider left lateral ventricle compared to the contralateral ventricle (Figure 1). Video electroencephalogram (EEG) showed a large number of high/extremely high amplitude spike-slow wave, multi-spike slow wave, and continuous release of slow wave during waking and sleeping periods. The patient’s developmental status assessment indicated a global developmental delay in all domains consisting of delay in cognition (HP: 0001263), language (HP: 0000750), fine motor, and gross motor (HP: 0002194). The clinical features of this patient are similar to those of Paganini-Miozzo syndrome which has been reported previously (Paganini et al., 2019; Sarmadian et al., 2023).

### 3.2 Mutation analysis by NGS

**FIGURE 2:**
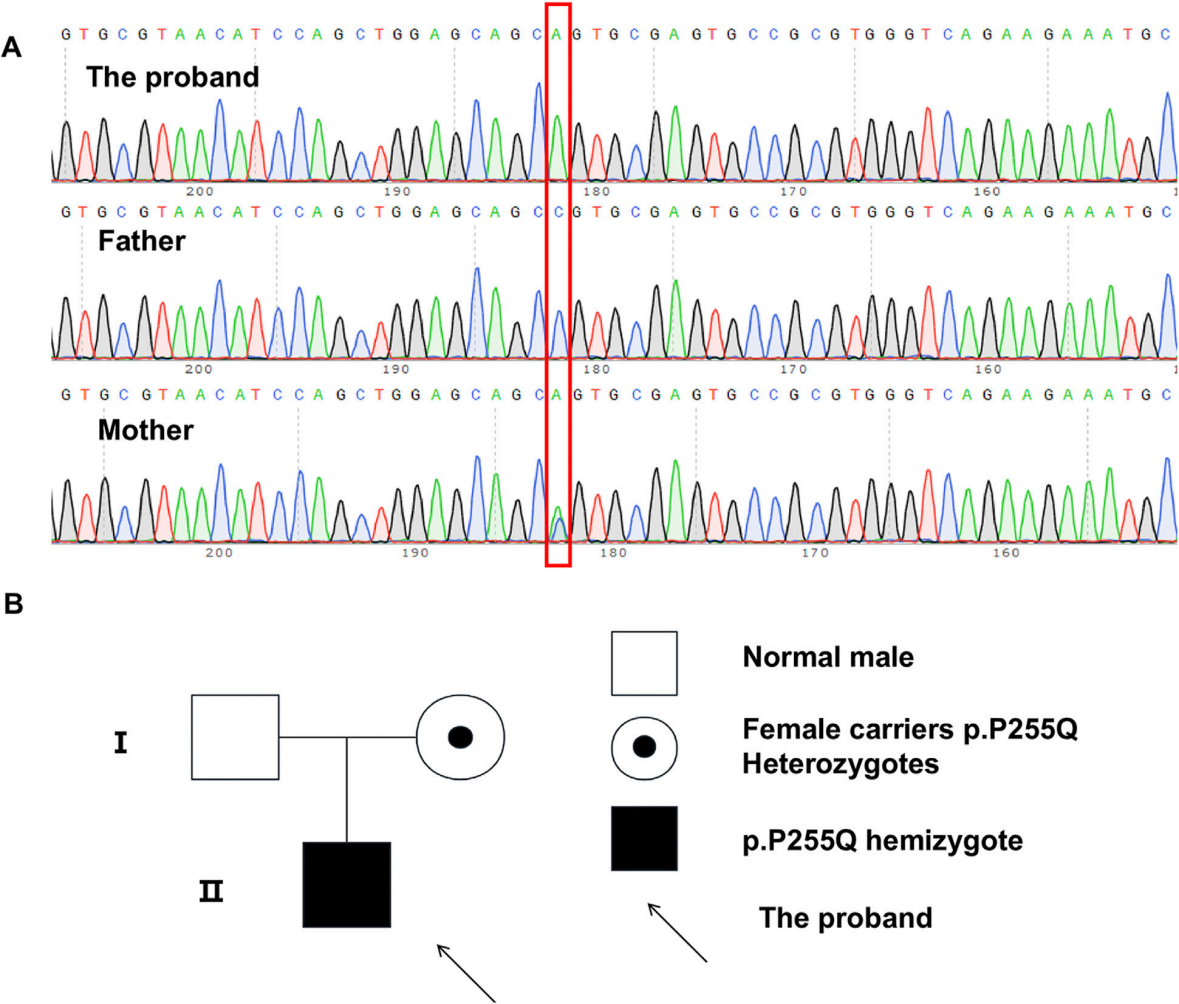
WES analysis and Pedigree of individuals carrying the HS6ST2 p.P255Q. **(A)** Sanger sequence results of proband and his parents. **(B)** Pedigree of individuals carrying the HS6ST2 p.P255Q.

Using WES analysis, we identified a novel hemizygous single-nucleotide variant, c.764C>A (p.Pro255Glu), in Paganini-Miozzo syndrome-related gene *HS6ST2* (NM_001077188.1, ENST00000521489.5) on chromosome Xq26.2 of the proband (Figure 2A). And this mutation has never been described before. Subsequently, we examined *HS6ST2* gene of the patient’s parents. The analysis results indicated the patient’s mother is a heterozygous carrier of the *HS6ST2* gene mutation c.764C>A p.Pro255Glu, whereas the father does not have this mutation (Figure 2B).

### 3.3 Effects of p.Pro255Glu variant on HS6ST2 protein structure

**FIGURE 3:**
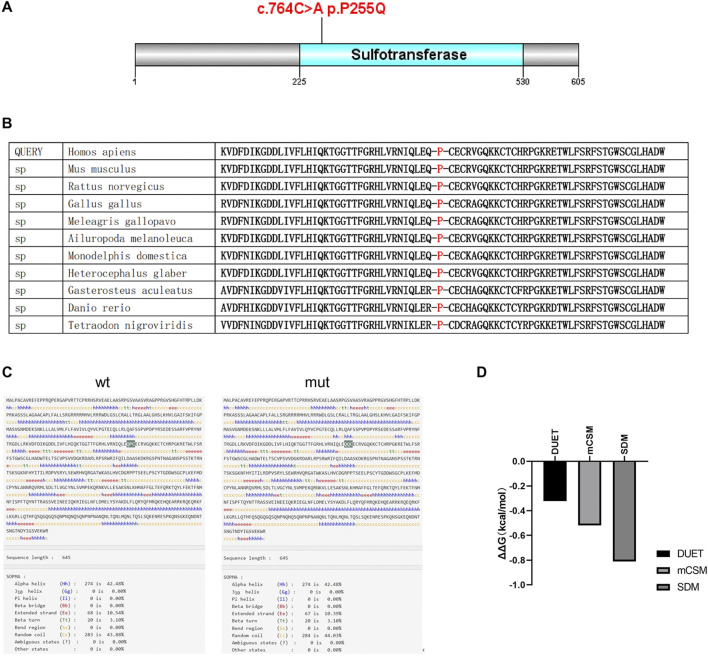
The effects of p.P255Q variant on HS6ST2 protein structure. **(A)** The amino acid sequence of HS6ST2 protein was analyzed using InterPro. The sulfotransferase catalytic activity domain is located at 225aa-531aa. And the c.764C>A p.P255Q mutation is located in the catalytic active region of sulfotransferase. **(B)** Analysis of the conservation of amino acid sequence of HS6ST2 protein. **(C)** The secondary structure of HS6ST2 protein was analyzed using SOPMA database. **(D)** Stability prediction of protein tertiary structure was conducted using three different algorithms, DUET, mCSM and SDM.

To explore the effects of P255Q variant on HS6ST2 protein structure and function, bioinformation analysis tools were used in this study. A portion of amino acid sequence of HS6ST2 protein was examined using Polymorphism Phenotyping v2 (PolyPhen-2). It was observed that the amino acid where the mutation c.764C>A p.P255Q occurs is conserved across multiple species, specifically being proline in 13 species (Figure 3B). The high conservation of this site suggests that it may play a crucial role in the function of HS6ST2. And then, InterPro was used to functionally characterize the HS6ST2 protein sequence. The results of InterProScan showed that the domain of HS6ST2 protein with sulfotransferase catalytic activity was located at 225aa-530aa, and the P255Q mutation was located exactly in this domain (Figure 3A). The secondary structure of HS6ST2 variant protein was analyzed using SOPMA database. According to the analysis results, the proportion of extended strand (Ee) and random coil (Cc) structures has changed after the mutation of HS6ST2 protein, and an Ee structure has become a Cc structure, indicating that the protein after mutation changes in secondary structure (Figure 3C). Considering that the stability of a protein is closely related to its biological activity, we made predictions about the stability of the mutant HS6ST2 protein. Three different algorithms, mCSM, SDM and DUET, were utilized. The calculation results of these algorithms all indicated significant changes in protein stability after mutation (Figure 3D). The predicted stability changes (ΔΔG) for the HS6ST2 protein were as follows: −0.519 kcal/mol (mCSM), −0.81 kcal/mol (SDM), and −0.321 kcal/mol (DUET). The negative ΔΔG values across all three algorithms suggest that the mutation reduces HS6ST2 protein stability. Notably, a ΔΔG below −0.5 kcal/mol (mCSM and DUET) or −1.0 kcal/mol (SDM) is indicative of high instability. Our findings indicate that the p.Pro255Glu site mutation moderately to severely destabilizes the HS6ST2 protein.

### 3.4 Effects of p.Pro255Glu variant on HS6ST2 expression

**FIGURE 4:**
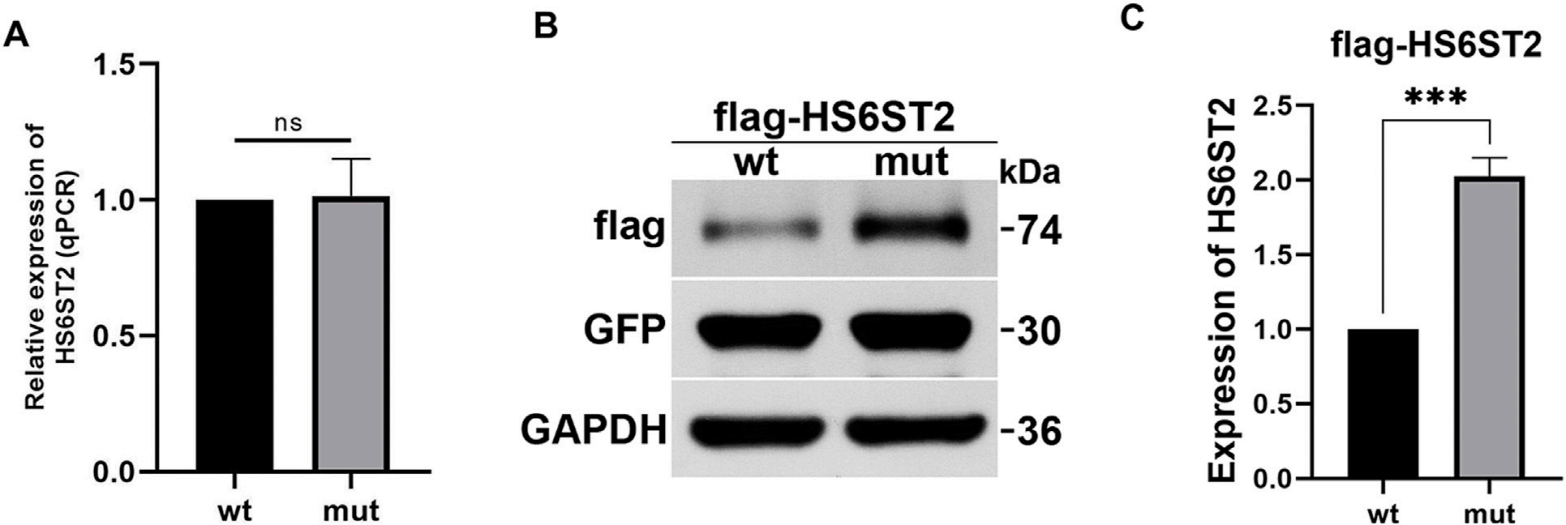
The effects of p.P255Q variant on HS6ST2 expression. HEK293 cells were transiently transfected with plasmids expressing wild-type flagHS6ST2 (wt), or mutant flag-P255Q (mut), and then total RNA and protein were extracted for qRT-PCR and Western blot. **(A)** The mRNA expressions of HS6ST2 wild type and its p.P255Q variant. **(B,C)** The protein expressions of HS6ST2 wild type and its p.P255Q variant. GFP served as the internal reference of transiently transfection. GAPDH served as the internal reference of Western blot. The t-test was used to compare the means of wild type group and variant group. ***P < 0.001, n.s., not significant.

To investigate the influence of P255Q variant on HS6ST2, plasmids expressing wild-type *HS6ST2* (HS6ST2-wt) or P255Q variant (HS6ST2-mut) were transiently transfected into HEK293T cells. Then the expressions of *HS6ST2* mRNA and protein were analyzed. As shown in Figure 4A, there was no significant difference between the wild-type and mutant in the expression level of mRNA. While, the expression of mutant HS6ST2 protein was increased dramatically comparing with the wild-type (Figures 4B,C). The result indicates that the effect of the mutant on HS6ST2 gene expression occurs at the post-transcriptional level rather than the transcriptional level.

## 4 Discussion

MRXSPM is a rare X-linked recessive genetic disease, with only three reported cases worldwide. The initial two cases were identified by Paganini et al. in identical twin brothers, aged 10, from Italy (Paganini et al., 2019). The third case was found in a 21-month-old boy from Iran (Sarmadian et al., 2023). This current case represents the fourth reported case globally and the first one reported in China. The proband is a 9-month-old boy, making him the youngest among the reported cases. All individuals affected by this disease exhibit hemizygosity for the X-linked gene *HS6ST2* mutation. Existing reports indicate that MRXSPM is predominantly associated with a clinical phenotype characterized by developmental defects, intellectual disability, abnormal vision, and mild facial deformity. All documented cases, including the present one, have exhibited noticeable developmental delay. The two earliest Italian cases were admitted due to intellectual disability. Both children began walking at the age of 2 and started talking at the age of 5. Their weight and height were significantly lower than the average growth and development of normal children. The Iranian case also experienced developmental delays. The 21-month-old Iranian boy was unable to sit or walk, and had difficulty expressing simple words clearly. In our case, the 9 months old proband was unable to actively turn over, sit firmly, crawl, or grasp objects. The child also had poor response to his name, limited eye contact, and showed clear signs of developmental delay. These observations indicate that developmental delay is one of the most prominent features of MRXSPM. Previous reports have documented cases of abnormal vision and facial deformities. The initial Italian twins exhibited high myopia, while the Iranian case and the proband in our case did not display any clinical signs of abnormal vision. But the previous three cases all displayed minor facial deformities, including the Iranian boy, whereas our case did not exhibit any such deformities (Table 1).

**TABLE 1:** Clinical features of the MRXSPM cases.

Clinical signs	Italian cases	Iranian case	Chinese case
Neurodevelopmental delay	+	+	+
Minor facial deformities	+	+	-
High myopia	+	Not mentioned	Not mentioned
Febrile seizures	+	+	Not mentioned
Brain CT	Lateral ventricular enlargement	Diffuse white matter in the periventricular, subarachnoid and juxtacortical areas	Lateral ventricular enlargement
EEG	Diffuse and irregular spikes	Burst suppression and hypsarrhythmia	Diffuse spinous slow wave
Glucose metabolism disorder	+	-	+
Liver function abnormality	Not mentioned	+	+
Elevated blood ammonia	Not mentioned	+	+

Considering the fact that MRXSPM is characterized by neurodevelopmental delay, all cases were subjected to brain CT and EEG examinations. The results of these examinations revealed the presence of brain abnormalities in these cases. The Italian twin cases exhibited febrile seizures before the age of 1, accompanied by epileptiform EEG abnormalities characterized by diffuse irregular spikes. Additionally, brain magnetic resonance imaging scans indicated mild lateral ventricular enlargement. The Iranian case demonstrated that epileptic seizures are primarily characterized by hand tremors. These seizures continue to occur frequently even after the child turns 1 year old. In contrast to the Italian cases, the brain MRI findings of Iranian case revealed more severe conditions, with extensive brain atrophy observed in the periventricular, subarachnoid, and paracortical areas. Additionally, there were diffuse white matter abnormalities in the brainstem (pons and posterior midbrain), putamen, cerebellum, and bilateral thalamus. The EEG results showed burst suppression and also detected irregular and inconsistent electrical activity with high amplitude, including slow, sharp waves and multifocal spikes, which manifested as peak rhythm disturbances. The results of this case are similar to those of the Italian case. The brain CT revealed mild lateral ventricular enlargement, and the EEG showed diffuse spinous slow wave (Table 1). According to the proband’s parents' description, there were no apparent symptoms of epilepsy in this case. However, it is important to note that this conclusion is solely based on the oral statement of the family members and requires further verification.

Based on the results of blood biochemical tests, there are notable differences among the aforementioned cases. Similar to the Italian cases, our case exhibited symptoms of glucose metabolism disorders, such as elevated blood lactate values and increased lactic acid/pyruvate values. However, the blood lactate levels of the two Italian children returned to normal after the age of 10. Regarding liver function, this case resembles the Iranian case in terms of elevated transaminase levels, but the increase observed in this study was greater. This indicates that the liver function abnormalities in the proband involved in this study are more pronounced. While, the liver function abnormalities has not been mentioned in previous cases in Italy (Table 1).

MRXSPM is caused by a male hemizygous mutation in the HS6ST2 gene located on chromosome Xq26. Through WES analysis, missense mutations in the HS6ST2 gene have been identified in the reported cases (Paganini et al., 2019; Sarmadian et al., 2023). The first reported Italian cases had a mutation of c.916G>C (p.Gly306Arg), and the Iranian case involved a mutation of c.979C>T (p.Pro327Ser) in the HS6ST2 gene. And a novel mutation of c.764C>A (p.Pro255Glu) was reported in the present study. As a member of the HS6ST family, HS6ST2 catalyzes the connection of sulfate to HSPG (Habuchi et al., 2000; Habuchi et al., 2003). HSPG binds to various proteins through its HS chain and regulates multiple signaling pathways (Bishop et al., 2007). Therefore, it plays a crucial role in cell growth, differentiation, adhesion, and migration (Song et al., 2011; Forsten-Williams et al., 2005). Research has demonstrated that HSPG is vital for visual development, motor neuron migration, and cranial axon development (Cai et al., 2014; Tillo et al., 2016). This also explains the occurrence of developmental delay in all MRXSPM cases and the presence of visual abnormalities in some cases.

Bioinformatics analysis suggests that the mutation site reported in our case is highly conserved across different species and may result in functional changes in the HS6ST2 protein. The variant c.916G>C (p.Gly306Arg) in the Italian case is predicted to modify the three-dimensional structure of the HS6ST2 enzyme, leading to alterations in its catalytic function and cellular localization. This prediction was further validated through subsequent enzyme activity tests. On the other hand, the variant c.764C>A (p.Pro255Glu) is predicted to impact the thermal stability of the HS6ST2 enzyme, consequently affecting its enzymatic activity. In addition, our findings indicate that this mutation can lead to an increase in intracellular HS6ST2 protein levels. This upregulation is observed at the post-transcriptional level, possibly due to an alteration in protein degradation rate caused by the mutation. Interestingly, the Italian case report did not show any impact of the mutant on HS6ST2 protein expression levels. These findings suggest that there might be variations in the mechanisms through which different mutants induce functional alterations in the HS6ST2 protein. Furthermore, these differences in mechanisms might explain the variations in clinical phenotypes observed across different cases.

Due to the limited number of reported cases of MRXSPM and the variability in mutation sites, it is currently challenging to comprehensively evaluate the defining characteristics of the disease. However, based on existing reports and this study, it is suggested that abnormal development of the nervous system and abnormal glucose metabolism are the primary manifestations of this genetic disease. Additionally, it may be accompanied by abnormalities in liver and kidney function, visual impairments, and facial deformities.

## Data Availability

The original contributions presented in the study are publicly available. This data can be found here: https://www.ebi.ac.uk/ena/browser/view/PRJEB90826.

## References

[B1] Bishop J. R. Schuksz M. Esko J. D. (2007). Heparan sulphate proteoglycans fine‐tune mammalian physiology. Nature. 446 (7139), 1030–1037. 10.1038/nature05817 17460664

[B2] Cai Z. Grobe K. Zhang X. (2014). Role of heparan sulfate proteoglycans in optic disc and stalk morphogenesis. Dev. Dyn. 243 (10), 1310–1316. 10.1002/dvdy.24142 24753163 PMC4177358

[B3] Forsten-Williams K. Chua C. C. Nugent M. A. (2005). The kinetics of FGF-2 binding to heparansulfate proteoglycans and MAP kinase signaling. J. Theor. Biol. 233, 483–499. 10.1016/j.jtbi.2004.10.020 15748910

[B4] Habuchi H. Miyake G. Nogami K. Kuroiwa A. Matsuda Y. Kusche-Gullberg M. (2003). Biosynthesis of heparan sulphate with diverse structures and functions: two alternatively spliced forms of human heparan sulphate 6‐O‐sulphotransferase‐2 having different expression patterns and properties. Biochem. J. 371 (Pt 1), 131–142. 10.1042/BJ20021259 12492399 PMC1223259

[B5] Habuchi H. Tanaka M. Habuchi O. Yoshida K. Suzuki H. Ban K. (2000). The occurrence of three isoforms of heparan sulfate 6-O-sulfotransferase having different specificities for hexuronic acid adjacent to the targeted N-sulfoglucosamine. J. Biol. Chem. 275, 2859–2868. 10.1074/jbc.275.4.2859 10644753

[B6] Jin Y. He J. Du J. Zhang R. X. Yao H. B. Shao Q. S. (2017). Overexpression of HS6ST2 is associated with poor prognosis in patients with gastric cancer. Oncol. Lett. 14 (5), 6191–6197. 10.3892/ol.2017.6944 29113266 PMC5661401

[B7] Paganini L. Hadi L. A. Chetta M. Rovina D. Fontana L. Colapietro P. (2019). A HS6ST2 gene variant associated with X-linked intellectual disability and severe myopia in two male twins. Clin. Genet. 95 (3), 368–374. 10.1111/cge.13485 30471091 PMC6392117

[B8] Sarmadian R. Gilani A. Biglari H. N. (2023). A novel variant of Paganini-Miozzo syndrome: a case report. Oxf Med. Case Rep. 3, omad024–omad116. 10.1093/omcr/omad024 PMC10041959 36993824

[B9] Song K. Li Q. Peng Y. B. Li J. Ding K. Chen L. J. (2011). Silencing of hHS6ST2 inhibits progression of pancreatic cancer through inhibition of Notch signalling. Biochem. J. 436, 271–282. 10.1042/BJ20110297 21443520

[B10] Tillo M. Charoy C. Schwarz Q. Maden C. H. Davidson K. Fantin A. (2016). 2‐ and 6‐O‐sulfated proteoglycans have distinct and complementary roles in cranial axon guidance and motor neuron migration. Development 143 (11), 1907–1913. 10.1242/dev.126854 27048738 PMC4920156

